# Optogenetic and chemogenetic strategies for sustained inhibition of pain

**DOI:** 10.1038/srep30570

**Published:** 2016-08-03

**Authors:** Shrivats M. Iyer, Sam Vesuna, Charu Ramakrishnan, Karen Huynh, Stephanie Young, Andre Berndt, Soo Yeun Lee, Christopher J. Gorini, Karl Deisseroth, Scott L. Delp

**Affiliations:** 1 Bioengineering, Stanford University, Stanford, CA 94305, USA; 2 Psychiatry and Behavioral Sciences, Stanford University, Stanford, CA 94305, USA; 3 Howard Hughes Medical Institute, Stanford University, Stanford, CA 94305, USA; 4 Mechanical Engineering, Stanford University, Stanford, CA 94305, USA.

## Abstract

Spatially targeted, genetically-specific strategies for sustained inhibition of nociceptors may help transform pain science and clinical management. Previous optogenetic strategies to inhibit pain have required constant illumination, and chemogenetic approaches in the periphery have not been shown to inhibit pain. Here, we show that the step-function inhibitory channelrhodopsin, SwiChR, can be used to persistently inhibit pain for long periods of time through infrequent transdermally delivered light pulses, reducing required light exposure by >98% and resolving a long-standing limitation in optogenetic inhibition. We demonstrate that the viral expression of the hM4D receptor in small-diameter primary afferent nociceptor enables chemogenetic inhibition of mechanical and thermal nociception thresholds. Finally, we develop optoPAIN, an optogenetic platform to non-invasively assess changes in pain sensitivity, and use this technique to examine pharmacological and chemogenetic inhibition of pain.

A recent comprehensive review of pharmacological management of neuropathic pain concluded that there remains “a substantial unmet need in patients with neuropathic pain” due to “inadequate response to drug therapy”^1^. This relative lack of efficacy in systemic pharmacological treatments for neuropathic pain is compounded by the significant negative consequences of addiction posed by prescription opioid pain-killers^2^,^3^. Spatially targeted, reversible silencing of primary afferent neurons has significant promise in the management of chronic pain^4^,^5^, and may represent a promising new class of treatments. Unlike systemic pharmacological therapy, such approaches would act directly at the injury locus without modulating the entire nervous system. Currently approved approaches to silence peripheral nerves are varied, and include the use of lidocaine patches, botulinum toxin injections, or high-dose capsaicin patches; however, evidence regarding their efficacy in treating chronic pain is limited^1^,^6^. These strategies indiscriminately block peripheral nerves and cannot specifically inhibit pain fibers while preserving functionality of other sensory fibers. Gene therapy approaches that modify neural excitability through constitutive expression or knockdown of synthetic or endogenous ion channels^7^, receptors, or peptides^8^ are under active development^9^,^10^, but do not permit tunable neuromodulation over time.

Two complementary approaches for reversible, stimulus-triggered neuromodulation have been developed over the past decade. The first, optogenetics, uses light as a stimulus to activate photosensitive targets to affect neural activity^4^,^11^,^12^,^13^,^14^. The second, chemogenetics, uses a small molecule ligand, (such as clozapine-N-oxide or recently characterized substitutes such as perlapine^15^) to activate synthetic G protein-coupled receptors (Designer Receptors Exclusively Activated by Designer Drugs, DREADDs) or ionic conductance (Pharmacologically Selective Actuator Modules, PSAMs), with varied downstream consequences on neuronal excitability^15^,^16^,^17^,^18^,^19^,^20^. While both are strong candidates for translation to human neuromodulation^4^,^5^,^18^,^19^,^21^,^22^, significant hurdles remain to be overcome.

In the optogenetic context, we and other groups have applied optogenetics to control peripheral neural circuits^23^,^24^,^25^,^26^,^27^,^28^,^29^,^30^,^31^, and have shown that transdermal illumination can be used to inhibit pain for a few seconds^28^,^29^,^30^; however, these efforts have required constant light, an impediment for clinical translation made clear by recent results demonstrating the consequences of high intensity illumination on local tissue heating^32^. Demonstrating that optogenetic inhibition can be achieved using intermittent light delivery is a critical feasibility barrier to use this technique on disease-relevant time scales.

Chemogenetic approaches to silencing peripheral nerves face no heating-related challenge; however, their ability to achieve behaviorally relevant inhibition of primary afferent nociceptors has not yet been demonstrated. In particular, the Gi-DREADD, hM4D(Gi), has been extensively used to enable chemogenetic silencing of neural circuits in the brain and spinal cord^16^,^17^,^33^, but has not been applied to control peripheral nociceptors.

Here, we describe two complementary strategies for sustained, reversible inhibition of specific sub-populations of primary afferent nociceptors. Using an intraneural viral injection approach, we express the step-function inhibitory channelrhodopsin (SwiChR^34^,^35^) in unmyelinated primary afferent nociceptors. This recently developed opsin enables light-triggered increases in cellular chloride conductance with slow off-kinetics, and has been reported to enable inhibition of neural projections in the brain without constant light^36^. Importantly, the SwiChR channel can be closed using red light, allowing in principle for precisely triggered induction and termination of optogenetic neuromodulation. Here, we demonstrate that it enables persistent inhibition of mechanical, thermal and formalin-induced nociception during post-illumination epochs. We characterize the time-profile of SwiChR enabled nociceptive inhibition, and demonstrate that SwiChR-induced inhibition can be sustained over long time-periods with temporally sparse illumination.

We then adapt the same viral expression strategy to express the hM4D(Gi) DREADD in primary afferent nociceptors and show that it enables inhibition of mechanical and thermal nociception. Finally, we develop optoPAIN (Optogenetic Pain Assay *in vivo*) to examine the complementarity of optogenetic and chemogenetic strategies to bidirectionally control pain without physically contacting the animal. We demonstrate that optoPAIN can be used to assess analgesic efficacy, and may have utility as a drug testing platform.

## Results

### SwiChR enables transdermal optogenetic inhibition of pain

**Figure 1:**
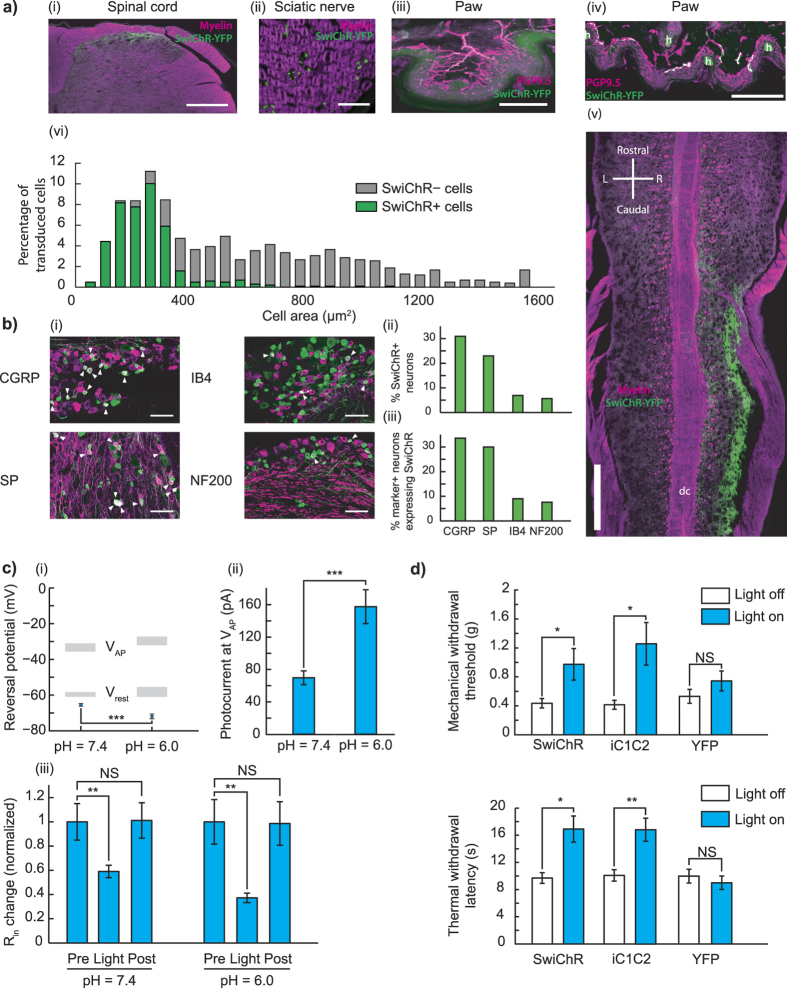
Intraneural injection of AAV6-hSyn-SwiChR-eYFP enables optogenetic inhibition of nociceptors during illumination *in vitro* and *in vivo*. (**a**) SwiChR-eYFP+ neurons project to i), v) lamina I/IIo in the spinal cord, are ii) unmyelinated, form free nerve endings in the iii) glabrous and iv) hairy paw and vi) are small in diameter. vi) Histogram based on 3 DRGs from 2 mice. *n* = 362 SwiChR+ neurons (green), *n* = 1078 SwiChR− neurons (grey). Scale bars: spinal cord (i): 250 μm, spinal cord (iv): 500 μm, nerve: 25 μm, paw (iii): 150 μm, paw (iv): 200 μm. Colors: i), ii), and v) magenta: myelin, green: SwiChR-eYFP. iii), iv) magenta: PGP9.5, green: SwiChR-eYFP. (**b**) i) Representative DRG sections showing overlap between SwiChR-eYFP and calcitonin gene-related peptide (CGRP), substance P (SP), isolectin B4 (IB4) binding neurons, and neurofilament-200 (NF200). Colors: green: SwiChR-eYFP, magenta: marker, white: overlap, arrowheads: co-expressing neurons. Scale bar: 100 μm. ii), and iii) Quantification, showing ii) percentage of SwiChR-eYFP+ neurons expressing a marker, and iii) percentage of neurons expressing a marker that co-express SwiChR-eYFP. Group data from >300 SwiChR-eYFP+ or marker + neurons from 3 different DRG sections from different mice. (**c**) i) Reversal potential of SwiChR relative to measured *V*_AP_ and *V*_rest_. (*P* = 0.0002, pH = 7.4: *n* = 14 cells for *V*_rev_ , *n* = 15 cells for *V*_AP_ , *n* = 21 cells for *V*_rest_ ; pH = 6.0: *n* = 8 cells for *V*_rev_ , *n* = 7 cells for *V*_AP_ , *n* = 9 cells for *V*_rest_). Gray zones: mean ± SEM. ii) Photocurrent amplitudes at *V*_AP_ . (*P* = 0.000174, pH = 7.4: *n* = 15 cells; pH = 6.0: *n* = 9). iii) Changes in cellular input resistance during and after blue light application normalized to pre-light value (pH = 7.4: *P* = 0.0018, *n* = 12 cells; pH = 6.0: *P* = 0.0050, *n* = 6 cells). (**d**) Mechanical and thermal thresholds and latencies increase significantly during blue-light illumination in SwiChR+ and iC1C2+ mice, but not YFP+ mice. *P < 0.05, **P < 0.01, ***P < 0.001.

We first asked whether SwiChR was functional *in vivo*, and could induce physiologically relevant inhibition during blue light illumination. We injected the sciatic nerves of female C57BL/6 mice with AAV6 vectors carrying SwiChR-eYFP under the control of the human synapsin-1 promoter. Two to three weeks following injection, we observed robust opsin expression that was restricted to unmyelinated small-diameter primary afferent neurons, projecting to lamina I/IIo of the spinal cord (Fig. 1a). We analyzed L4 and L5 dorsal root ganglion sections to evaluate the overlap of SwiChR-eYFP expression and cellular markers for nociceptive and non-nociceptive primary afferent neurons. SwiChR-eYFP+ primary afferent neurons primarily expressed peptidergic nociceptive markers, and minimally overlapped with neurofilament-200, a marker for large-diameter neurons (Fig. 1b). Results were consistent with those we have previously observed following intrasciatic injection of AAV6-hSyn-ChR2-eYFP^28^, and indicate strong expression of SwiChR throughout the primary afferent nociceptor.

We then recorded from dissociated cultured dorsal root ganglia (DRGs) obtained from AAV6-hSyn-SwiChR-eYFP injected mice. Reversal potential and photocurrent amplitude measurements demonstrated that electrophysiological properties of the SwiChR channel recorded from DRGs were indistinguishable from those previously recorded *in vitro* in hippocampal culture^34^ (Fig. 1c). We observed that SwiChR was responsive to a blue light pulse, and induced significant decreases in input resistance during illumination (Fig. 1c). Consistent with previous reports^34^, reducing pH negatively shifted the SwiChR reversal potential and increased SwiChR photocurrent (Fig. 1c). This is of particular relevance as the skin is known to have reduced pH relative to the rest of the body, indicating that SwiChR-mediated photocurrents may be particularly potent in the peripheral projections of primary afferent neurons.

Next, we examined how transdermally delivered blue light affected nociceptive assays. We performed blinded mechanical threshold assays on mice expressing SwiChR, YFP, or the chloride-conducting inhibitory channelrhodopsin iC1C2. Blue light produced large, statistically significant increases in mechanical withdrawal thresholds (SwiChR+ mice: *P* = 0.047, *n* = 10 mice, 123% increase. iC1C2+ mice: *P* = 0.033, *n* = 10 mice, 203% increase. YFP mice: *P* = 0.37, *n* = 9 mice) and thermal latency measures (SwiChR+ mice: *P* = 0.014, *n* = 10 mice, 61% increase. iC1C2+ mice: *P* = 0.0078, *n* = 9 mice, 67% increase. YFP+ mice: *P* = 0.69, *n* = 6 mice) in iC1C2+ and SwiChR+ mice, but not in YFP+ mice (Fig. 1d). Thermal withdrawal latency assessed using a modified Hargreaves apparatus, as described in previous work^28^. The degree of inhibition we observed here was comparable to that we have previously reported in halorhodopsin (eNpHR3.0) expressing mice^28^.

**Figure 2:**
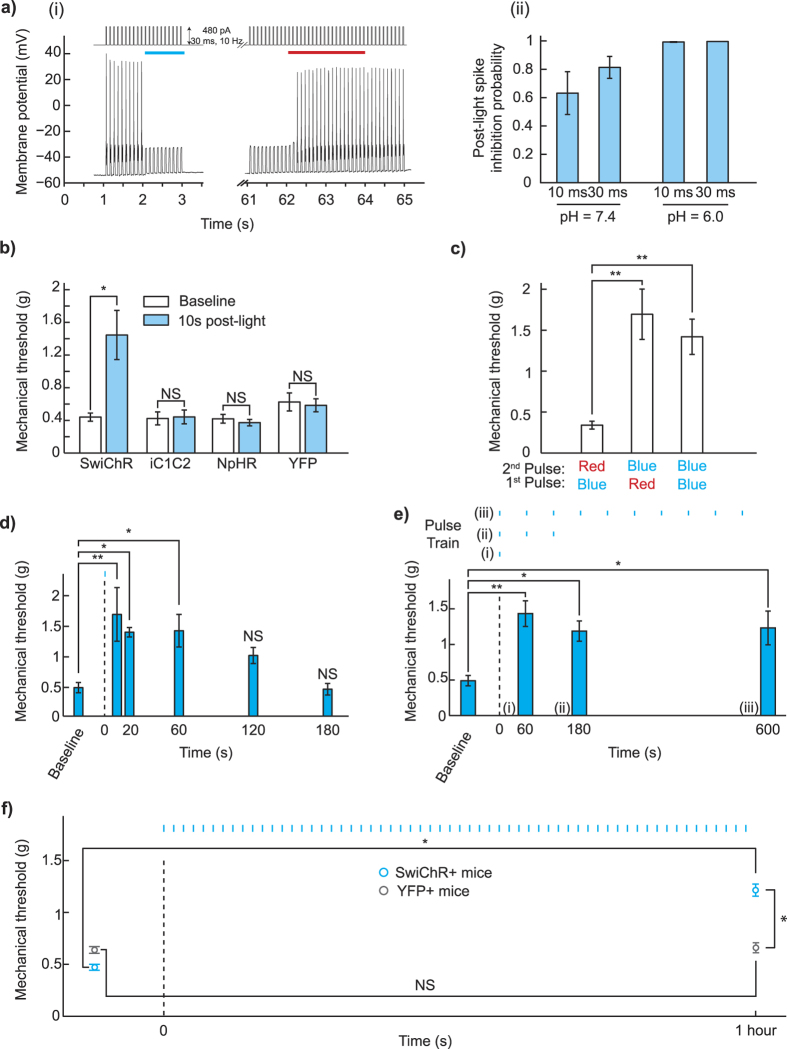
SwiChR inhibits nociceptor-driven pain after illumination (**a**) i) Voltage traces from SwiChR+ neurons stimulated with pulsed current injection (480 pA, 10 Hz, 30 ms) ii) Probability that an action potential is inhibited at 60 seconds after blue light application in the protocol shown in (**b**) i), using 10 Hz current injection (pH = 7.3, 10 ms pulses: 297.1 ± 43.8 pA, *n* = 11 cells; 30 ms pulses: 256.2 ± 33.8 pA, *n* = 15 cells; pH = 6.0, 10 ms pulses: 374.4 ± 85.1 pA, *n* = 8 cells; 30 ms pulses: 507.1 ± 72.5 pA, *n* = 8 cells). (**b**) Mechanical thresholds measured in the absence of blue light and 10 seconds after light application. (**c**) Mechanical thresholds measured in SwiChR+ mice after a two-pulse sequence of different colors of light. (**d**) Mechanical thresholds measured in SwiChR+ mice at time-points after a single blue light pulse. (**e**) Mechanical thresholds measured in SwiChR+ mice after trains of blue light pulses (1 s pulses, 1/60 Hz). Response to a i) single pulse at *t* = 60 s, ii) 3 pulses at *t* = 180 s, and iii) 10 pulses at *t* = 600 s. (**f**) Mechanical thresholds measured before and after a one hour train of blue light pulses (1 s pulses, 1/60 Hz). *P < 0.05, **P < 0.01, ***P < 0.001.

We turned then to examining the ‘post-light’ period, in which inhibitory effects are not seen in experiments conducted with halorhodopsins or archaerhodopsins^37^. In cultured, SwiChR + DRG neurons, we observed that a single 1 second blue light pulse was sufficient to induce inhibition of electrically evoked action potentials not only during the light pulse, but also for many seconds following, with high spike inhibition probabilities observed as late as 60 seconds after light stimulus (Fig. 2a). As expected^34^,^35^, optogenetic inhibition could be rapidly terminated through illumination with red light (Fig. 2a), which causes the SwiChR channel to close. Consistent with results described in Fig. 1c, the efficiency of SwiChR-mediated post-light inhibition increased with reductions in pH (Fig. 2a).

### SwiChR-mediated inhibition can be sustained through sparse illumination

We then examined whether this ‘post-light’ inhibition property of SwiChR was meaningfully retained *in vivo*, enabling optogenetic inhibition during the ‘post-light’ period. We tested mechanical thresholds in a blinded fashion 10 seconds after a brief blue light pulse, and observed increases in withdrawal threshold only in SwiChR+ mice, and not in iC1C2+, eNpHR3.0+, or YFP+ mice (Fig. 2b, SwiChR+ mice: *P* = 0.015, *n* = 7 mice, 228% increase. iC1C2+ mice: *P* = 0.89, *n* = 7 mice. NpHR+ mice: *P* = 0.49, *n* = 10 mice, YFP+ mice: *P* = 0.79, *n* = 7 mice). Remarkably, inhibition of pain in SwiChR+ mice appeared equivalently effective in the ‘post-light’ state as in the ‘light-on’ state. Consistent with known properties of the step-function mutation, and recent reports using SwiChR in the medial prefrontal cortex^36^, SwiChR-mediated inhibition during the ‘light-off’ period could be terminated on demand using a pulse of red light (Fig. 2c, a Red-Blue light-sequence produced higher thresholds than a Blue-Red sequence (*P* = 0.0043, *n* = 7 mice, 399% increase), as did a Blue-Blue sequence (*P* = 0.0015, *n* = 7 mice, 318% increase).

We then explored the temporal dynamics of SwiChR-mediated post-light inhibition to determine its feasibility for long-term inhibition. We illuminated the mouse paw with a blue light pulse (1 s), and observed the effect on mechanical thresholds at various times after the light pulse. Mechanical thresholds were significantly and stably higher than baseline when measured 1 minute after the light pulse, consistent with electrophysiological recordings from cultured DRG neurons (Fig. 2a,d). Thresholds returned to baseline by 3 minutes after the single light pulse (One-way ANOVA: *F*(5, 18) = 5.21. *P* = 0.0039, *n* = 4 mice at each time-point. Dunnett’s test: *P*(*t* = 10 s) = 0.006, *P*(*t* = 20 s) = 0.0415, *P*(*t* = 60 s) = 0.0355, *P*(*t* = 120 s) = 0.3587, *P*(*t* = 180 s) ≈ 1).

We tested whether appropriately timed supplementary light pulses could be used to extend the duration of SwiChR-mediated inhibition. We illuminated mice with 1 second blue light pulses, delivered once per minute. Optogenetic inhibition was stable with this temporally sparse illumination paradigm; mechanical thresholds remained significantly higher than baseline 3 minutes and 10 minutes after the first light pulse (Fig. 2e, One-way ANOVA: *F*(3, 20) = 5.19. *P* = 0.0082, *n* = 6 mice for each pulse-train. Dunnett’s test: *P*(*t* = 60 s) = 0.004, *P*(*t* = 180 s) = 0.0337, *P*(*t* = 600 s) = 0.0231). We observed that even after 1 hour of temporally sparse blue light pulses, SwiChR+ mice showed stably raised pain thresholds that were statistically indistinguishable from raised thresholds observed after a single blue light pulse (*P* = 0.55, *n* = 7 in each group). YFP+ mice showed no significant change in mechanical thresholds (Fig. 2f, SwiChR+ mice: *P* = 0.014, *n* = 7 mice, 166% increase. YFP+ mice: *P* = 0.9, *n* = 10 mice. SwiChR+ mice versus YFP+ mice after light: *P* = 0.012, *n* as earlier, SwiChR+ post-light thresholds are 86% higher than YFP+ post-light thresholds).

Finally, in a pilot experiment, we explored the potential for sparse illumination as a strategy to control non-reflexive pain-related behavior. The formalin test is a commonly used pain assay, phase I of which is primarily driven by direct activation of nociceptors^38^, phase II of which is driven in part by inflammatory and spinal facilitation mechanisms^39^,^40^. We injected mice in the plantar surface of the paw with a 4% formalin solution, and then placed them in an apparatus where they received blue light illumination once every minute (Supp. Fig. 1). Consistent with working hypotheses regarding the formalin test, we observed that SwiChR+ mice, but not YFP+ mice showed reduced pain behavior in phase I of the test (Phase I: *P* = 0.029, *n* = 5 mice each, 83% decrease), while no significant difference between the two groups was seen in phase II of the test (Phase II: *P* = 0.41, *n* = 5 mice each)., indicating that optogenetic inhibition of transduced unmyelinated primary afferents was sufficient to reduce nociceptor-triggered Phase I pain behavior, but was insufficient to mitigate the broader inflammatory response observed in phase II. These results indicate that step-function inhibitory opsins can enable meaningful optogenetic inhibition over experimentally relevant time-periods. Future studies that exploit the genetic specificity of optogenetics will help to examine whether this differential response holds when different subsets of primary afferent neurons are optogenetically inhibited.

### Chemogenetic strategies increase mechanical and thermal pain thresholds

**Figure 3:**
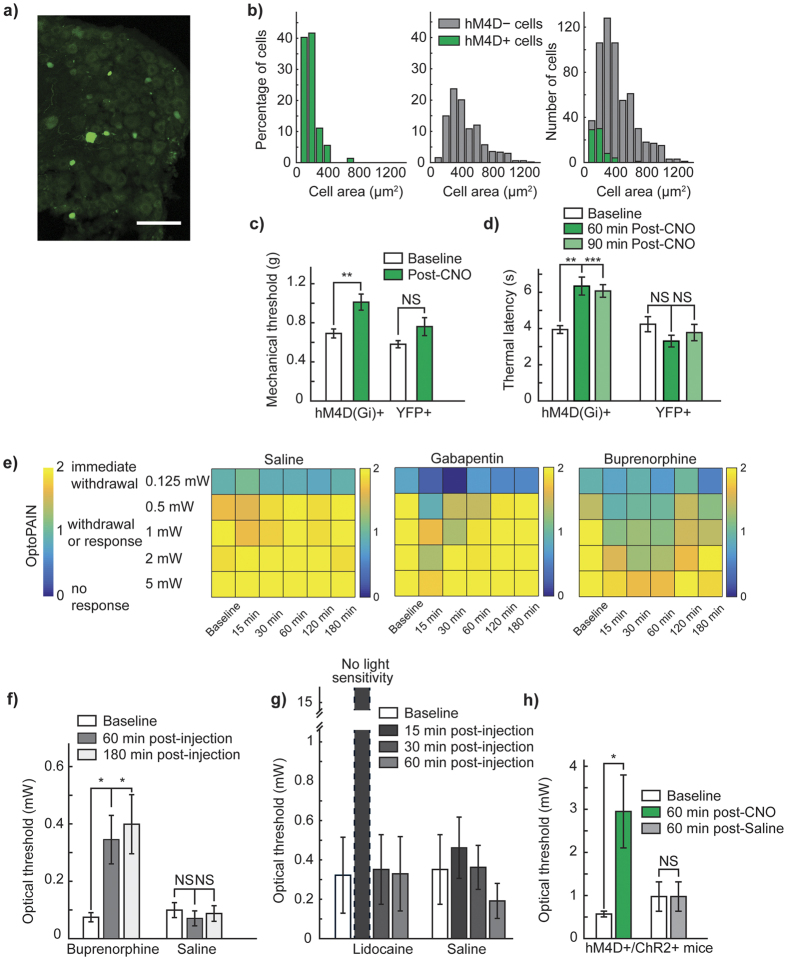
Chemogenetic inhibition of pain and the OptoPAIN assay (**a**) Representative DRG from mouse injected intraneurally with AAV6-hSyn-HA-hM4D(Gi)-IRES-mCitrine. Scale bar: 100 μm. (**b**) Histogram of size-distribution of hM4D+ (green) and hM4D− (gray) cells, expressed as relative percentage. (**c**) Mechanical thresholds in hM4D+ and YFP+ mice following CNO administration. (**d**) Thermal thresholds in hM4D+ and YFP+ mice following CNO administration. (**e**) Qualitative light-sensitivity scores showing differential response to various administered agents (saline: *n* = 5 mice, gabapentin: *n* = 3 mice, buprenorphine: *n* = 4 mice). (**f**) Light-intensity thresholds following intraperitoneal injection of buprenorphine (25 mg/kg) or saline (100 μl). (**g**) Light-intensity thresholds following intraplantar injection of lidocaine (20 μl of 2% lidocaine), or saline (20 μl). (**h**) Light-intensity thresholds in mice co-injected with AAV6::ChR2 and AAV6::hM4D following intraperitoneal injection of CNO or saline. *P < 0.05, **P < 0.01, ***P < 0.001. Bonferroni correction applied in Fig. 3d,f, with significance level 0.025.

Chemogenetic strategies have been used to chronically modulate G-protein coupled receptors *in vivo* over time-periods as long as 4 weeks^41^ and may therefore be a suitable option for reversible stimulus-triggered inhibition of pain. We intraneurally injected mice with AAV6-hSyn-HA-hM4D(Gi)-IRES-mCitrine, and observed mCitrine expression in small-diameter nociceptors (Fig. 3a,b). Expression patterns were consistent with those previously observed with AAV6 mediated expression of SwiChR.

We examined the effect of intraperitoneal clozapine-N-oxide (CNO) administration on hM4D+ mice pain thresholds. In blinded experiments, we observed that CNO robustly increased mechanical withdrawal thresholds in mice expressing the hM4D receptor (Fig. 3c, hM4D+ mice: *P* = 0.0059, *n* = 12 paws, 45.3% increase, YFP+ mice: *P* = 0.064, *n* = 12 paws). The effect size of inhibition we observed was comparable to that we have previously reported with optogenetic inhibition of primary afferents using NpHR (0.802)^28^. When we turned to chemogenetic inhibition of thermal sensation, we observed similarly strong inhibition of Hargreaves thresholds. Following CNO administration, we observed a 61% increase in thermal withdrawal latency at 60 minutes post-injection, which was maintained as a 54% increase as late as 90 minutes post-injection (hM4D+ mice: 60 min: *P* = 0.00092, 61% increase, 90 min: P = 0.00041, 54% increase, *n* = 10 paws, YFP+ mice: 60 min: *P* = 0.045, *P* = 0.26, *n* = 10 paws, significance threshold at 0.025 due to Bonferroni correction). The effect size of the inhibition observed was similar to that previously observed with optogenetic inhibition of thermal perception using NpHR (2.05)^28^.

### Optogenetic assays of nociception

We were curious if the degree of inhibition induced by Gi-DREADD activation would be sufficient to affect optogenetic activation of pain-related responses. To assess this, we developed a threshold light-intensity assay to non-invasively measure changes in pain state (OptoPAIN), and validated that this assay was meaningfully affected by known analgesics (Fig. 3e,f, buprenorphine: 60 min: *P* = 0.022, 180 min: *P* = 0.022, saline: 60 min: *P* = 0.46, 180 min: *P* = 0.76, *n* = 4 mice throughout) and local anesthetics (Fig. 3g, lidocaine: 15 min: no light sensitivity, 30 min: *P* = 0.42, 60 min: *P* = 0.42, saline: 15 min: *P* = 0.42, 30 min: *P* = 0.96, 60 min: *P* = 0.29, *n* = 3 mice throughout). Administration of analgesic or local anesthetic agents, but not saline, resulted in both qualitative (Fig. 3e, Supplementary Fig. 2), as well as quantitative (Fig. 3f,g) changes in light-sensitivity across light intensities. We then intraneurally injected mice with a mixture of AAV6-hSyn-ChR2-YFP and AAV6-hSyn-HA-hM4D-IRES-mCitrine. In blinded experiments, we observed that following CNO administration, the required light intensity to achieve a pain-related response increased by 418%, indicating that while the optogenetic stimulatory effect was ultimately stronger than the chemogenetic inhibitory effect, chemogenetic inhibition could significantly modulate optogenetic sensitivity (CNO: *P* = 0.045, *n* = 5 mice, 417.5% increase, saline: *P* ≈ 1, *n* = 5 mice).

## Discussion

These results are the first *in vivo* demonstration of sustained optogenetic inhibition of pain over long time periods, a meaningful step towards clinical translation. SwiChR appears to inhibit peripheral neural circuits as effectively as traditional inhibitory pumps, and does so with a 98% reduction in the required duration of light exposure. The robust nature of step function opsin-mediated inhibition we observe may be linked to the low pH of the skin^42^, which may increase the photocurrent, and strengthen the inhibition obtained at the free nerve ending through transdermal illumination. As described in a Behavioral Note in the Methods section, we conducted all SwiChR-related behavioral experiments in dimly lit areas following a preparatory pulse of red light, due to our concern that ambient light may be sufficiently bright as to trigger superficial SwiChR+ free nerve endings. Further improvements in the light-sensitivity, reversal potential, photocurrent, and time kinetics of inhibitory chloride-conducting channelrhodopsins^35^, as well as transdermal light delivery devices, are likely to increase the utility of this approach of sparse illumination for chronic inhibition of neural circuits. A remaining challenge for both optogenetic and chemogenetic approaches to inhibiting peripheral circuits is persistent transgene expression – the development of solutions for long-term expression in peripheral nerves will be essential to translation of these techniques^43^.

Additionally, optoPAIN, an all-optical pain threshold assay, demonstrates the utility of the primary afferent nociceptor system as a readily accessible neural circuit for screening pain states and opsin neuromodulation *in vivo*^14^,^28^,^31^. Peripheral nerves can be rapidly transduced, non-invasively manipulated in freely moving, untethered animals, and excitation or inhibition produces easily observable behavioral readouts. We show that this assay captures the analgesic effects and known temporal dynamics of analgesic drugs such as buprenorphine. As animals tested in this way do not need to be touched physically, optoPAIN may allow for pain measurement during complex behaviors such as social interaction, memory retrieval, attention and drug seeking with less potential for experimenter-induced bias^44^.

While optogenetic strategies inherently require illumination, a manipulation with little clinical precedent, systemic delivery of chemogenetic ligands with local effects on neural activity is compatible with clinical practice^19^. Open questions regarding the efficacy of these methods in humans remain, but seem promising given recent experiments in non-human primates^45^,^46^. Demonstrating peripheral nerve opsin and DREADD transduction in non-human primates, and meaningful peripheral nerve inhibition via transdermal light patches or orally delivered chemogenetic ligands would substantially advance these technologies as a new class of pain management strategies.

## Methods

### Animal subjects and experiments

The procedures described here were approved by the Stanford APLAC, and were carried out in accordance with APLAC and NIH guidelines for care and use of laboratory animals. Age-matched female C57BL/6 mice were randomly assigned by cage to control and experimental groups.

### Viral injections

Vectors used: AAV6-hSyn-eYFP (3 × 10^12^ vg/ml), AAV6-hSyn-biC1C2-TS-eYFP (4.4 × 10^13^ vg/ml) (AAV6:iC1C2), AAV6-hSyn-biC1C2(C128A)-TS-eYFP (1.9 × 10^13^ vg/ml) (AAV6:SwiChR), AAV6-hSyn-ChR2-eYFP (2.4 × 10^13^ vg/ml), AAV6-hSyn-HA-hM4D-IRES-mCitrine (2.3 × 10^13^ vg/ml), and AAV6-hSyn-eNpHR3.0-eYFP (1.6 × 10^13^ vg/ml). All vectors were ordered from the UNC Vector Core. Intraneural injections of a total of 5 μl of undiluted virus were performed as previously described^28^. Briefly, following induction of anesthesia (2% isoflurane), sterilization of the surgical site, and infusion of local anesthetic (100 μl of 0.25% Bupivacaine), the sciatic nerve was exposed through blunt dissection of the connective tissue between the gluteus superficialis and the biceps femoris muscles. While minimizing nerve manipulation, a 35G needle was inserted under the epineurium of the nerve, and the virus injection performed at 1 μl/min, using a 25 μl syringe (Hamilton Company) connected to a Harvard PHD Syringe pump (Harvard Apparatus). Two separate injections of 2.5 μl were performed into the common peroneal and tibial branches of the sciatic nerve. In the case where AAV6::hM4D and AAV6::ChR2 were injected, 2.5 μl of each undiluted virus was injected in a 5 μl bolus, as before in two separate injections into each nerve branch. Following injection, the incision was sutured closed using 5-0 silk suture. All behavioral testing was performed at 2–5 weeks following viral injection.

### Culture and electrophysiology of DRG neurons

Mice were anesthetized three to five weeks after intraneural injection, and transcardially perfused with 10 ml of 4 °C PBS. Lumbar DRGs were removed and placed in 4 °C sterile MEM-complete solution (minimal essential media, MEM vitamins, antibiotics and 10% FBS). DRGs were desheathed and transferred to MEM-Collagenase solution, incubated for 45 min in a water bath (37 °C) and triturated in 2.5 ml TrypLE Express (Invitrogen). The trypsin was quenched with 2.5 ml MEM-complete with 2.5 mg/ml MgSO_4_, 100 μg/ml trypsin inhibitor from chicken egg white and 80 μg/ml DNase I. Cells were centrifuged and resuspended in MEM-complete at a cell density of 500,000 cells/ml. 100 μl of the suspension was placed on matrigel-coated coverslips, and incubated at 37 °C C, 3% CO_2_, 90% humidity. Two hours after incubation, neurons were flooded with 1 ml of MEM-complete. Cells were maintained in culture for 3 days prior to electrophysiology.

#### Electrophysiology

We used a Spectra X Light engine (Lumencor) coupled to the fluorescence port of an Olympus BX61WI microscope to image and deliver light. Light power density through a 40X objective was measured with a power meter (ThorLabs), and 475/15 and 632/22 filters were used for blue light (3 mW/mm^2^) and red light (10.6 mW/mm^2^) respectively.

We followed recording procedures identical to those previously described^34^. The internal solution recording solution contained (in mM): 140 K-gluconate, 10 HEPES, 10 EGTA, 2 MgCl2, pH 7.3. The external recording solution contained (in mM): 135 NaCl, 4 KCl, 10 HEPES, 2 CaCl2, 2 MgCl2, 30 D-glucose, pH 7.3, 12 Citric acid/Na-Citrate, pH 6.0, with synaptic transmission blockers 25 μM D-APV, 10 μM NBQX. Recordings were made using a MultiClamp700B amplifier (Molecular Devices). Measurements were corrected for the liquid junction potential of +15.5 mV. pClamp10.3 (Molecular Devices), OriginLab8 (OriginLab), and Sigmaplot (SPSS) software was used to record and analyze data.

Upon light activation, the stationary photocurrent was used as photocurrent amplitude. Action potential threshold was measured at the voltage deflection point at which the first-order derivative of the membrane potential (dV/dt) exhibited a sharp transition, typically >10 mV/ms. Input resistance was calculated from the steady-state current responses evoked by 10 mV hyperpolarizing steps in voltage-clamp mode. A red light pulse was applied before all recordings.

To investigate action potential inhibition, spikes were evoked with intracellular current injections (10 ms and 30 ms pulse widths, 90–850 pA, at 10 Hz). A blue light was applied for 1 s during current injection. Spike inhibition probability was calculated as the fraction of electrically evoked spikes blocked during the light pulse epoch. Red light was applied for 1 s, 60 seconds after the blue light pulse to recover spiking.

### Optogenetic mechanical withdrawal threshold testing

#### Experimental procedure

Mice were habituated to the testing apparatus prior to testing. The room was dimly lit with a diffuse red light during habituation (3–10 μW/mm^2^). Von Frey hairs were applied to the plantar surface of the paw using the up-down method^47^. After fiber application, a red light was flashed below the mouse illuminating the plantar surface. For experiments involving SwiChR+, iC1C2+, or YFP+ mice, a blue laser (473 nm, 1–8 mW/mm^2^, OEM Laser Systems) was shone on the plantar paw during or before fiber application for light-on and post-light experiments. For post-light experiments involving NpHR+ and YFP+ mice, a yellow laser (593 nm, 1–8 mW/mm^2^, OEM Laser Systems) was used. When pulse-trains were used, pulse-width was 1 s, frequency 1/60 Hz, and a custom fabricated LED floor (475 nm, Cree) was used to illuminate the paw. Experimenters were blinded as to mouse identity in Figs 1d, 2b,f and 3c–h. Mechanical withdrawal threshold testing was performed by a single (male) tester^44^.

#### Red-Blue testing

We measured mechanical sensitivity after the following light patterns: blue then blue, red then blue, blue then red. Light pulses were separated by 60 seconds, the von Frey fiber was presented 60 seconds after the final light.

#### Statistics

In the light-on, 10 s post-light, Red-Blue, and one-hour experiments, changes in threshold were assessed using a two-sided paired Student’s *t*-test. The SwiChR+ and YFP+ groups in the one-hour experiment were tested for homoscedasticity using the Levene’s test, then a two-sided unpaired, homoscedastic Student’s *t*-test was used. In tests conducted at multiple time-points, a one-way ANOVA was used. The Dunnett’s post-hoc multiple comparisons test was used to determine which time-points were significantly different from baseline.

### Optogenetic thermal withdrawal latency testing

We measured thermal sensitivity under blue light illumination using a previously described modified Hargreaves apparatus^28^. Mice were habituated to the testing chamber for 20 minutes under a diffuse red light (3–10 μW/mm^2^). A blue LED-ring emitting 1–3 mW was placed around the infrared emitter to illuminate the paw during testing. The experimenter was blinded as to mouse identity. Note that to allow for placement of the blue LED-ring the Hargreaves apparatus floor was raised, resulting in higher baseline latencies.

### Behavioral note

Throughout these experiments, we observed that mediated inhibition was most reliable when animals were placed in dimly lit areas. We believe that this may be due to the high light-sensitivity SwiChR exhibits and the superficial nature of the fibers transduced. As an additional experimental precaution, mice received red-light stimulation prior to any blue light experiments to ensure SwiChR channels began in a closed state.

### Immunohistochemistry, imaging and quantification

#### Tissue sections

Mice were anesthetized and transcardially perfused with 10 ml, 4 °C PBS, and 10 ml, 4 °C paraformaldehyde (4%, PFA). Mouse spinal cords, sciatic nerves, paws, and lumbar dorsal root ganglia were dissected, fixed in 4% PFA overnight, and cryoprotected in 30% sucrose, at 4 °C. Tissue was frozen in O.C.T. (Tissue-Tek), cut into 20–40 μm thick sections on a cryostat (Leica CM3050S), and mounted on slides (Superfrost). All DRG, paw, nerve, and transverse spinal cord sections were cut at 20 μm, while longitudinal spinal sections were performed at 40 μm to enable easier mounting of tissue.

#### Immunohistochemistry

We followed procedures identical to those previously described elsewhere^28^. Primary antibodies used were Rabbit anti-NF200 (1:100, N4142, Sigma-Aldrich), Rabbit anti-CGRP (1:5000, C8198, Sigma-Aldrich), Rat anti-Substance P (1:500, 556312, BD Pharmingen), Biotin-IB4 (1:50, B-1205, Vector Laboratories), Rabbit anti-GFP (1:500, ab290, Abcam) and Rabbit anti-PGP9.5 (1:500, CL31A3, Cedarlane). Secondary antibodies used were Donkey anti-Rabbit Cy5 (1:500, 711-175-152, Jackson Laboratories), Donkey anti-Rat Cy5 (Cy3 Donkey anti-Rat (1:500, 712-175-153), and Streptavidin Texas Red (3:100, SA-5006, Vector Laboratories). For myelin or cell-size quantification, we used FluoroMyelin Red (1:300 in PBS, F34652, Molecular Probes) and NeuroTrace Nissl stain (1:500, 21482, Life Technologies) respectively.

#### Imaging

Slides were imaged with a Leica TCS SP5 confocal scanning laser microscope using 20× and 40× oil immersion objectives. Images were processed using Fiji^48^, and image brightness and contrast were adjusted when required. If such adjustments were made, they were made uniformly to the entire image.

#### Quantification

To quantify SwiChR+ cell-size, 2 DRG sections each from 3 mice were stained using the NeuroTrace stain, cell outlines traced and area quantified. To quantify hM4D+ cell-size, 2–3 DRGs from 2 mice were stained with anti-GFP antibodies, cell outlines traced, and area quantified. To quantify nociceptive and non-nociceptive marker overlap, sections from 1 DRG each from 3–4 different mice were imaged, and SwiChR+, marker+, and overlapping cells counted. All quantification was performed on L4 and L5 DRGs, and was performed on the DRG section with the greatest visible cross-sectional area of DRG cell bodies.

### OptoPAIN assay methods

We used two complementary approaches to assess the effect of analgesics on mouse light-sensitivity. In all cases, mice initially received intra-neural injections of AAV6-hSyn-ChR2-eYFP. Experiments were conducted 3–4 weeks following injections. In all cases, mice were habituated to the test chamber for 30 min, were randomly assigned to receive intraperitoneal injections of ‘test compound’, or saline, and the observer was blinded as to injection identity.

In the first, qualitative approach, mice were exposed to three successive flashes of blue light (473 nm, delivered transdermally through a fiber optic cable), with varying total exposed intensity (0.125 mW, 0.5 mW, 1 mW, 2 mW, 5 mW), and the averaged response noted. Mouse responses to pain were coded as 0, 1, or 2, based on the following rubric: 0: no pain response, 1: ambiguous pain response (spreading of paw, increased attention to paw, movement of unclear origin), or 2: clear pain response (paw flinch, mouse licking or illuminated paw). Mice were initially tested to obtain baseline sensitivity values, test agents (Buprenorphine: 100 μl, 0.25 mg/kg. Gabapentin: 100 μl, 100 mg/kg, Saline: 100 μl) were then administered intraperitoneally with brief isoflurane anesthesia. Mouse sensitivity was assayed at 5 time-points post-injection (15 min, 30 min, 60 min, 120 min, and 180 min).

In the second, more quantitative approach, mouse sensitivity was assayed by analogy to the ‘up-down’ method for von Frey testing. Mouse sensitivity to a given light intensity was taken as a binary metric (pain-sensitive vs. not pain-sensitive). Mice were initially tested at 0.5 mW. If mice showed a positive pain-response, then a lower intensity illumination was tested, if a negative pain-response was seen, then a higher intensity illumination was tested, until a 50% response-inducing light intensity could be determined. As a positive control, we also performed an intra-plantar injection of lidocaine, and tested whether light sensitivity was reversibly blocked. Light intensities used were: 0.02 mW, 0.05 mW, 0.125 mW, 0.25 mW, 0.5 mW, 1 mW, 2 mW, 5 mW and 10 mW. Mouse sensitivity was assayed at various time-points post intraperitoneal injection (Buprenorphine: 100 μl, 0.25 mg/kg. Saline: 100 μl) or post intra-plantar injection (Lidocaine: 20 μl of 2% lidocaine).

In the combination chemogenetic-optogenetic experiments, mice received injections of a mixture of AAV6::hM4D and AAV6::ChR2, (2.5 μl of each undiluted virus was injected in a 5 μl bolus), and the second method was used to assess chemogenetic inhibition, at 60–90 min after injection of CNO (100 μl, 10 mg/kg).

### Chemogenetic assays

#### Mechanical withdrawal testing

Mice were tested 3–5 weeks after intraneural injection. Mice were placed on a von Frey apparatus, and allowed to habituate for 1 hour. Mechanical withdrawal thresholds were obtained using the up-down method of testing. Mice were then briefly anesthetized with isoflurane, and injected intraperitoneally with 100 μl of clozapine-N-oxide (for an effective dose of 10 mg/kg) or 100 μl of saline. Post-injection testing was done in a blinded manner, with mechanical withdrawal thresholds collected at 45–75 minutes post-injection. Data were then unblinded, and statistical significance assessed using the Student’s *t*-test.

#### Thermal withdrawal testing

Mice were tested 3–5 weeks after intraneural injection. Mice were placed on a Hargreaves apparatus, and allowed to habituate for 1 hour. Thermal withdrawal latencies were then obtained. Mice were then briefly anesthetized with isoflurane, and injected intraperitoneally with 100 μl of clozapine-N-oxide (for an effective dose of 10 mg/kg) or 100 μl of saline. Post-injection testing was done in a blinded manner, with thermal withdrawal latencies collected at 45–75 minutes post-injection. Data were then unblinded, and statistical significance assessed using the Student’s *t*-test.

### Group data

Throughout, group data is shown as Mean ± SEM, and (*) represents *P* < 0.05, (**) represents *P* < 0.01, and (***) represents *P* < 0.001, N.S. denotes ‘not significant’, *P* > 0.05.

## Additional Information

**How to cite this article**: Iyer, S. M. *et al*. Optogenetic and chemogenetic strategies for sustained inhibition of pain. *Sci. Rep.* **6**, 30570; doi: 10.1038/srep30570 (2016).

## Supplementary Material

Supplementary Information
