# Supplementary material for: Optogenetic and chemogenetic strategies for sustained inhibition of pain
Source: Sci Rep. 2016 Aug 3;6:30570. doi: 10.1038/srep30570 (PMC4971509; doi:10.1038/srep30570)
Supplement: Supplementary Information [file srep30570-s1.doc]

**Title:** Optogenetic and chemogenetic strategies for sustained inhibition of pain – Supplementary Information

**Authors:** Shrivats M. Iyer^1*^, Sam Vesuna^1*^, Charu Ramakrishnan^1^, Karen Huynh^1^, Stephanie Young^1^, Andre Berndt^1^, Soo Yeun Lee^1^, Christopher J. Gorini^1^, Karl Deisseroth^1,2,3^, Scott L. Delp^1,4^

**Affiliations:** ^1^Bioengineering, ^2^Psychiatry and Behavioral Sciences, ^3^Howard Hughes Medical Institute, ^4^Mechanical Engineering, Stanford University.

*These two authors contributed equally to this paper.

**Conflicts of Interest:** S.M.I. and S.L.D. have filed a patent application related to methods described in this paper.

**Corresponding author:** Scott L. Delp; James H. Clark Center S321, 318 Campus Drive, Stanford, CA 94305, [delp@stanford.edu](mailto:delp@stanford.edu), 650-723-1230.

**Supplementary Figure Legends**

Supplementary Figure 1: SwiChR+ mice spend significantly less time licking than YFP+ mice in Phase I, but not in Phase II of the test. Phase I: *P* = 0.029, *n* = 5 mice each, 83% decrease. Phase II: *P* = 0.41, *n* = 5 mice each.

Supplementary Figure 2: Quantified data for Fig. 3e. Intraperitoneal administration of Gabapentin (100 mg/kg), and Buprenorphine (0.25 mg/kg), but not Saline causes reduced OptoPAIN scores that exhibit both time and light-intensity dependence.

**Supplementary Figure 1**

**
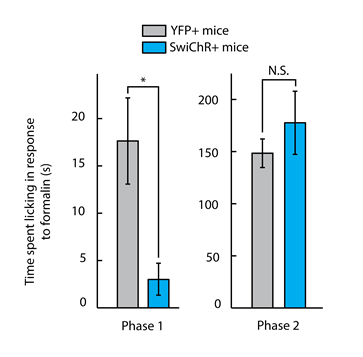
**


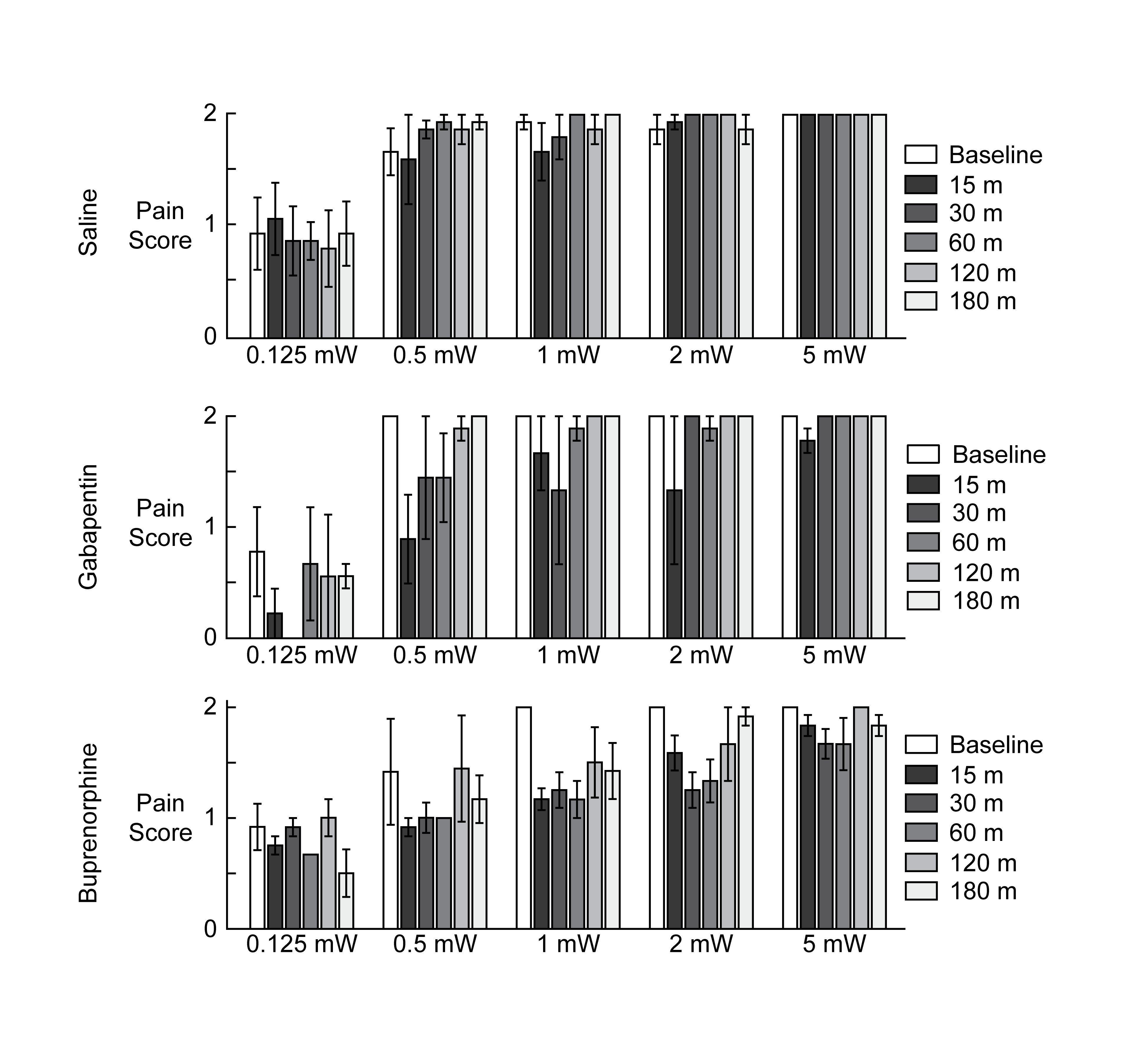
**Supplementary Figure 2**
